# Necroptosis Underlies Hepatic Damage in a Piglet Model of Lipopolysaccharide-Induced Sepsis

**DOI:** 10.3389/fimmu.2021.633830

**Published:** 2021-03-12

**Authors:** Qiao Xu, Junjie Guo, Xiangen Li, Yang Wang, Dan Wang, Kan Xiao, Huiling Zhu, Xiuying Wang, Chien-An Andy Hu, Guolong Zhang, Yulan Liu

**Affiliations:** ^1^ Hubei Key Laboratory of Animal Nutrition and Feed Science, Wuhan Polytechnic University, Wuhan, China; ^2^ Department of Biochemistry and Molecular Biology, University of New Mexico School of Medicine, Albuquerque, NM, United States; ^3^ Department of Animal and Food Sciences, Oklahoma State University, Stillwater, OK, United States

**Keywords:** necroptosis, liver injury, sepsis, necrostatin-1, pigs, lipopolysaccharide

## Abstract

**Background:**

Necroptosis is a newly recognized form of programmed cell death with characteristics of both necrosis and apoptosis. The role of necroptosis in hepatic damage during sepsis is poorly understood. In this study, we investigated the occurrence of necroptosis in hepatic damage, and its contribution to hepatic damage in a piglet model of lipopolysaccharide (LPS)-induced sepsis.

**Methods:**

Two animal experiments were conducted. In trial 1, piglets were challenged with LPS and sacrificed at different time points after LPS challenge. In trial 2, piglets were pretreated with necrostatin-1, a specific inhibitor of necroptosis, prior to LPS challenge. Alterations in the hepatic structure and function, pro-inflammatory cytokine expression, and the necroptosis signaling pathway were investigated. Typical ultrastructural characteristics of cell necrosis was observed in the liver of LPS-challenged piglets.

**Results:**

Expressions of critical components of necroptosis including kinases (RIP1, RIP3, and MLKL), mitochondrial proteins (PGAM5 and DRP1), and an intracellular damage-associated molecular pattern (HMGB1) were increased in the liver in a time-dependent manner, followed by hepatic inflammation, morphological damage, and dysfunction as manifested by elevated hepatic expression of IL-1β, IL-6 and TNF-α as well as increased serum AST and AKP activities and the AST/ALT ratio. Pretreatment with necrostatin-1 significantly reduced the expression of RIP1, RIP3 and MLKL as well as PGAM5, DRP1 and HMGB1, which subsequently led to obvious attenuation of hepatic inflammation and damage.

**Conclusions:**

Our study demonstrates that necroptosis occurs in the liver during sepsis and contributes to septic hepatic injury.

## Introduction

Sepsis is a common and life-threatening illness that could lead to multiple organ failure, shock, and death as a result of an exaggerated inflammatory response to infection (1, 2). As a main cause of deaths in intensive care units, sepsis accounts for an estimated 200,000 deaths annually in the US alone (3). The liver is a target tissue frequently damaged by sepsis (4). Severe septic liver damage may further cause multiple organ dysfunction syndrome (5). It is important to understand the mechanism of septic hepatic injury before devising effective therapeutic strategies.

Hepatic cell death is a critical event in progression of various liver diseases (6, 7). Traditionally, apoptosis and necrosis are recognized as two main modes of cell death contributing to the pathogenesis of liver diseases (7). In recent years, necroptosis, a novel mode of cell death, has been identified (8, 9). Necroptosis incorporates features of both necrosis and apoptosis, and is strictly regulated by receptor interacting protein kinase (RIP) 1 and 3 (9, 10). Auto- and transphosphorylations of RIP1 and RIP3 are required for assembly of RIP1/3-containing necrosome and activation of necroptotic signaling (9, 11). RIP3 subsequently recruits and phosphorylates mixed lineage kinase domain-like protein (MLKL) to promote its oligomerization and translocation to plasma membrane, resulting in membrane rupture and necrotic cell death (9). In addition, the RIP1/3- containing necrosome has also been reported to activate the mitochondrial phosphatase phosphoglycerate mutase family member 5 (PGAM5), which recruits and activates the mitochondrial fission factor dynamin-related protein 1 (DRP1) (12, 13). Activation of DRP1 results in mitochondrial fragmentation and reactive oxygen species generation, resulting in necrotic cell death (14). Cell rupture and necrosis lead to the release of intracellular damage-associated molecular patterns (DAMP) such as high-mobility group box 1 (HMGB1) protein, which triggers inflammation and secondary tissue damage (15).

Accumulating evidence has indicated that necroptosis plays a crucial role in several forms of liver injury in mice such as alcoholic liver disease (16), non-alcoholic fatty liver disease (17) and liver ischemia-reperfusion injury (18). However, the role of necroptosis in septic liver injury is rarely investigated. Pig is an excellent model for human physiology and pathology (19, 20). In the present study, we employed a piglet model of LPS-mediated sepsis (21, 22), to confirm the occurrence of necroptosis in the liver. The contribution of necroptosis to liver damage was further explored by pretreatment of animals with necrostatin-1 (Nec-1), a well-described inhibitor of necroptosis (23), prior to LPS challenge.

## Methods

### Animals and Experimental Design

Animal experimental procedures were approved by the Animal Care and Use Committee of Wuhan Polytechnic University (Wuhan, China). A total of 70 weaned, apparently healthy piglets (Duroc × Large White × Landrace, 28 ± 3 d, average body weight of 7.1 ± 0.9 kg) were purchased from Aodeng Agricultural and Animal Husbandry Technology Co., Ltd (Hubei, China). The pigs were individually housed in 1.80 × 1.10 m^2^ pens, maintained in an environmentally controlled room, and received a standard weanling piglet diet for acclimation for 14 d with free access to feed and water. Animals were observed daily for signs of diarrhea, sickness, and abnormal behavior.

In the first experiment, 42 weanling piglets were randomly allocated to seven groups including a control group and six LPS groups slaughtered at six different time points. Each group had six piglets. The piglets in LPS groups were injected intraperitoneally with *Escherichia coli* LPS (*Escherichia coli* serotype 055: B5, MilliporeSigma, St. Louis, MO, USA) at 100 μg/kg body weight (BW) and then were slaughtered at 1, 2, 4, 8, 12, or 24 h after LPS challenge (**Table 1S**). The piglets in the control group were slaughtered right (0 h) after injection with an equivalent volume of sterile saline. The dose of LPS administered was shown to trigger an obvious inflammatory response and sepsis based on our earlier experiments (22, 24).

In the second experiment, 28 weanling piglets were randomly assigned to four groups in a 2 × 2 factorial design with seven piglets in each group. The animals were pretreated intraperitoneally with Nec-1 (MedChem Express, NJ, USA) at 1.0 mg/kg BW or an equivalent volume of 2% DMSO 30 min before intraperitoneal injection of LPS at 100 μg/kg BW or saline (**Table 2S**). The piglets were killed at 4 h after LPS or saline injection.

### Blood and Liver Sample Collection

Prior to animal sacrifice, blood samples were collected into 10-mL vacuum tubes (Becton Dickinson Vacutainer Systems; Franklin Lakes, NJ, USA) *via* jugular vein puncture, and centrifuged at 3,500 g for 10 min to collect serum. Serum was stored at –80°C until analysis. Following blood collection, the pigs were humanely euthanized with an intramuscular injection of sodium pentobarbital (80 mg/kg BW), and then the liver samples (0.5 cm^3^ segments) were harvested. One portion of the liver samples was fixed in fresh 4% paraformaldehyde/phosphate-buffered saline for 24 h for histological analysis. Another portion was cut into smaller pieces (about 1 mm^3^) and fixed with 2.5% glutaraldehyde at 4°C for ultrastructural transmission electron microscopy (TEM) analysis. The remaining liver tissue was diced into smaller pieces, snap frozen in liquid nitrogen, and stored at −80°C for further analysis.

### Biochemical Measurements of the Serum Samples

The activities of alanine aminotransferase (ALT), aspartate aminotransferase (AST) and alkaline phosphatase (AKP) in the serum were determined using a colorimetric method with commercial kits (Nanjing Jiancheng Biological Product, Nanjing, China) according to the manufacturer’s instructions.

### Liver Histological Analysis

After a 24-h fixation, liver segments (5-mm) were dehydrated with a series of increasing concentrations of ethanol, cleared with xylene, and embedded in paraffin. Cross-sections (4-μm thick) of each sample were deparaffinized, and stained with hematoxylin and eosin. Histological analysis was performed in a blinded manner by an experienced pathologist using a light microscope with a computer-assisted morphometric system (BioScan Optimetric, BioScan, Edmonds, WA, USA).

### Liver Ultrastructural Analysis

Liver samples were fixed with 2.5% glutaraldehyde, and postfixed in 1% osmium tetroxide. They were then dehydrated with ethanol and embedded in Epon 812 (Eimicon, Shanghai, China). Ultra-thin sections (100 nm) were cut and stained with uranyl acetate and lead citrate. Ultrastructural observation was conducted in a blinded manner by an experienced pathologist using a transmission electron microscope (TEM) (Tecnai, FEI, Hillsboro, USA) at an accelerating voltage of 200 kV and a magnification of 5,000.

### Measurement of Proinflammatory Cytokine Concentrations

Frozen liver samples were weighed, homogenized in nine volumes of ice-cold saline solution, and centrifuged at 2,500 rpm for 10 min at 4°C to collect the supernatants. The protein concentrations of the supernatants were measured using the bicinchoninic acid reagent (Applygen Technologies Co., China). The concentrations of tumor necrosis factor-α (TNF-α), interleukin (IL)-6 and IL-1β in the supernatants were determined using respective ELISA kits (R&D Systems, Minneapolis, MN, USA) according to the manufacturer’s protocols and expressed as pg/mg protein.

### Protein Expression Analysis by Western Blot

Liver samples (100-150 mg) were suspended in lysis buffer, homogenized, and centrifuged to collect the supernatants. Protein concentrations of the supernatants were measured using the bicinchoninic acid reagent (Applygen Technologies Co., China). Equal amounts of hepatic proteins were loaded and separated on a polyacrylamide gel and transferred onto polyvinylidene difluoride membranes. Immunoblots were blocked for at least 1 h with 3% bovine serum albumin in TBS/Tween-20 buffer. The membranes were incubated overnight at 4°C with primary antibodies and then with the horseradish peroxidase-labelled secondary antibodies for 2 h at room temperature. Specific primary antibodies included rabbit anti-RIP1 (#LS-B8214, LifeSpan), rabbit anti-RIP3 (#SC-135170, Santa Cruz), rabbit anti-phosphorylated MLKL (p-MLKL) (#62233, Cell Signaling), rabbit anti-PGAM5 (*#*ab131552, Abcam), rabbit anti-DRP1 (*#*ab154879, Abcam), rabbit anti-HMGB1 (#PAB12414, Abnova), and mouse anti-β-actin (#A2228, Sigma Aldrich). Blots were developed using an Enhanced Chemiluminescence Western blotting kit (Amersham Biosciences, Sweden), visualized using a Gene Genome Bioimaging System, and analyzed using GeneTools software (Syngene, Frederick, MD, USA). The relative protein abundance of target proteins were expressed as the ratio of target protein/β-actin protein.

### mRNA Expression Analysis by Real-Time PCR

Total RNA was isolated from the liver samples by using TRIzol (TaKaRa Biotechnology Co., Dalian, China) and quantified using Nano-Drop 2000 Spectrophotometer (Thermo Scientific, Wilmington, DE, USA). RNA purity was assessed by determining the ratio of the absorbance at 260 and 280 nm, and the integrity was verified using agarose gel electrophoresis. Genomic DNA removal and cDNA synthesis were performed using PrimeScript^®^ RT Kit (TaKaRa Biotechnology). Real-time PCR analysis was performed using a SYBR^®^ Premix Ex Taq™ (Tli RNaseH Plus) qPCR kit (TaKaRa Biotechnology) on an Applied Biosystems 7500 Real-Time PCR System (Applied Biosystems, Life Technologies). The total volume of each PCR reaction was 20 μL, which contained 10.0 μL SYBR^®^ Premix Ex Taq^TM,^ 0.4 μL forward primer (10 μmol/L), 0.4 μL reverse primer (10 μmol/L), 0.4 μL ROX reference dye II (10×), 2.0 μL cDNA and 6.8 μL RNase-free water. After an initial denaturation at 95°C for 30 s, the reaction was cycled at 95°C for 5 s and 60°C for 34 s for 40 times. The primer pairs used are shown in **Table 1**. The expression level of each target gene relative to a reference gene (GAPDH) was analyzed using the 2^-ΔΔCt^ method of Livak and Schmittgen (25). Our results demonstrated that GAPDH did not exhibit any variation among treatments. Relative mRNA abundance of each target gene was expressed as the fold change of its expression level in an experimental sample over that in control samples.

**Table 1:** Primer sequences used in real-time PCR.

Gene	Forward (5’-3’)	Reverse (5’-3’)
TNF-α	AAGACACCATGAGCACTGAGA	CGACCAGGAGGAAGGAGAAG
IL-1β	GCTAACTACGGTGACAACAATAATG	CTTCTCCACTGCCACGATGA
IL-6	AAGGTGATGCCACCTCAGAC	TCTGCCAGTACCTCCTTGCT
RIP1	ACATCCTGTACGGCAACTCT	CGGGTCCAGGTGTTTATCC
RIP3	CTTGTTGTCTGTCCGTGAGC	GAGGAGGTTGGGCTGTTGA
MLKL	TCTCGCTGCTGCTTCA	CTCGCTTGTCTTCCTCTG
PGAM5	TCTTCATCTGCCACGCCAAT	GGTGATGCTGCCGTTGTTG
DRP1	TGTGGGCTGCAGGTCATTA	TTGCGCTGGGACATTTTAGC
HMGB1	GCCTATCCATTGGTGATGTTG	TCCTCCTCCTCCTCCTCAT
GAPDH	CGTCCCTGAGACACGATGGT	GCCTTGACTGTGCCGTGGAAT

DRP1, dynamin-related protein 1; HMGB1, high-mobility group box 1; IL, interleukin; MLKL, mixed-lineage kinase domain-like protein; PGAM5, phosphoglycerate mutase family member 5; RIP, receptor interacting protein kinase;TNF-α, tumor necrosis factor-α.

### Statistical Analysis

In the first experiment, the data were analyzed using Duncan’s multiple comparison tests. In the second experiment, the data were analyzed using the general linear model procedures of ANOVA (SAS, Cary, NC, USA). Post hoc testing was conducted using Duncan’s multiple comparison tests. All data were presented as means ± standard errors of the mean. *P* ≤ 0.05 was considered statistically significant.

## Results

### Hepatic Damage and Dysfunction Are Dynamically Induced in LPS-Challenged Piglets

**Figure 1:**
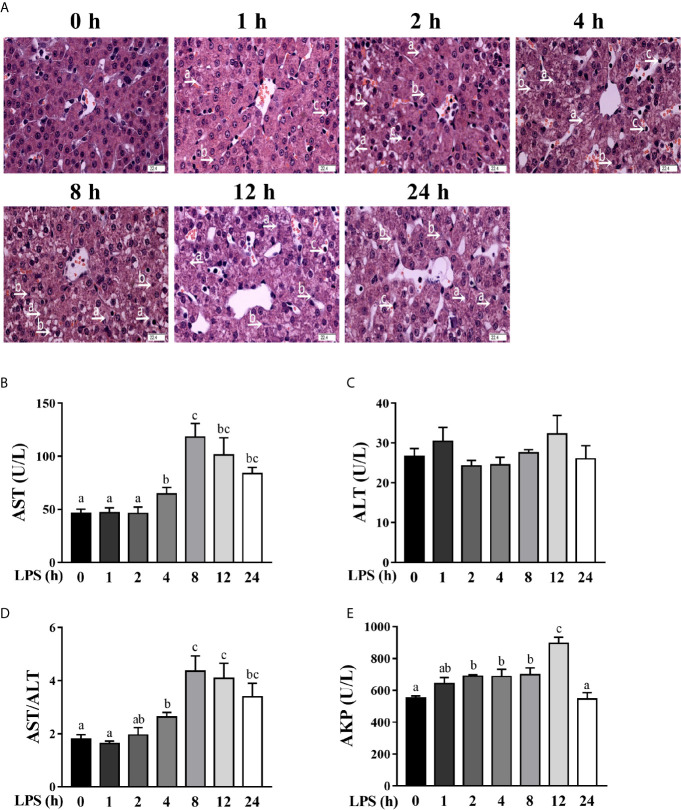
Hepatic damage and dysfunction are dynamically induced in LPS-challenged piglets. Piglets were sacrificed right after injection with sterile saline and served as controls (0 h), while others were challenged with LPS at 100 μg/kg body weight and sacrificed at 1, 2, 4, 8, 12, or 24 h after challenge. **(A)** Representative haematoxylin/eosin-stained liver sections of the control (Con) group and LPS groups. Caryolysis (a) and karyopycnosis (b) of hepatocytes, inflammatory cell infiltration (c), disordered hepatic cell cord arrangement, and hepatocyte vacuolation were observed in LPS-challenged samples. Magnification: 400 ×, scale bars = 22.4 μm. **(B–E)** Serum AST, ALT and AKP activities and the AST/ALT ratio. Values are means ± SEM, n = 6. ^abc^ Means without a common letter differ significantly (*p* < 0.05).

To investigate the dynamic effect of LPS on the liver, we examined hepatic histopathological changes of pigs at 1, 2, 4, 8, 12, and 24 h after LPS challenge. Compared to the control pigs (0 h), LPS-challenged pigs displayed a progressive increase in liver damage as manifested by disordered hepatic cell cord arrangement, inflammatory cell infiltration, and karyolysis, karyopyknosis, and vacuolation of hepatocytes (**Figure 1A**). Hepatic injury gradually increased between 1-4 h, became severe between 4-12 h and alleviated obviously at 24 h.

To explore the dynamic effect of LPS challenge on hepatic function, we measured the activities of AST, ALT and AKP in the serum (**Figures 1B–E**), which are commonly used makers of hepatic function (22, 24). LPS challenge increased serum AST activity between 4-24 h (peaking at 8 h), the AST/ALT ratio between 4-24 h (peaking at 8 h), and the AKP activity between 2-12 h (peaking at 12 h) (*p* < 0.05). However, LPS had no effect on serum ALT activity.

### Hepatic Inflammation Is Dynamically Induced in LPS-Challenged Piglets

**Figure 2:**
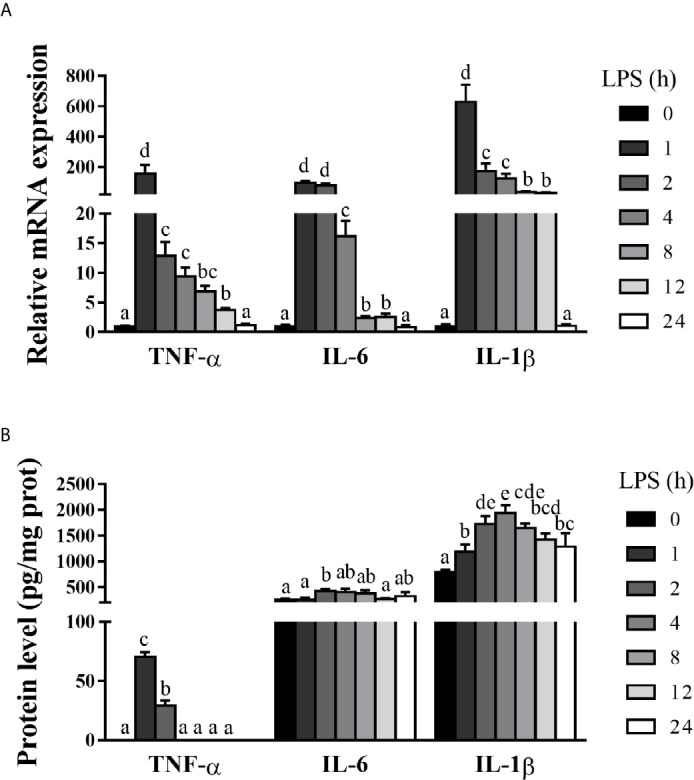
Hepatic inflammation is dynamically induced in LPS-challenged piglets. The piglets in the control group were sacrificed at 0 h after injection with sterile saline, while those in LPS groups were sacrificed at 1, 2, 4, 8, 12, or 24 h after challenge with LPS at 100 μg/kg body weight. **(A)** TNF-α, IL-1β and IL-6 mRNA expression in the liver. **(B)** TNF-α, IL-1β and IL-6 protein levels in the liver. Values are means ± SEM, n = 6. ^abcde^ Means without a common letter differ significantly (*p* < 0.05).

Hepatic inflammation contributes to hepatic damage (22, 24). To investigate the dynamic effect of LPS on hepatic inflammatory response of pigs, we analyzed mRNA and protein levels of hepatic pro-inflammatory cytokines including TNF-α, IL-6 and IL-1β (**Figures 2A, B**). LPS challenge triggered a marked, but temporary increase of these pro-inflammatory cytokines. Specifically, mRNA expression levels of TNF-α, IL-6, and IL-1β were elevated between 1-12 h, peaked at 1 h, and returned to the basal levels at 24 h. Similarly, LPS elevated protein concentrations of TNF-α between 1-2 h (peaking at 1 h), IL-6 at 2 h, and IL-1β between 1-24 h (peaking at 4 h) (*p* < 0.05).

### Hepatic Cell Necroptosis Is Dynamically Induced in LPS-Challenged Piglets

**Figure 3:**
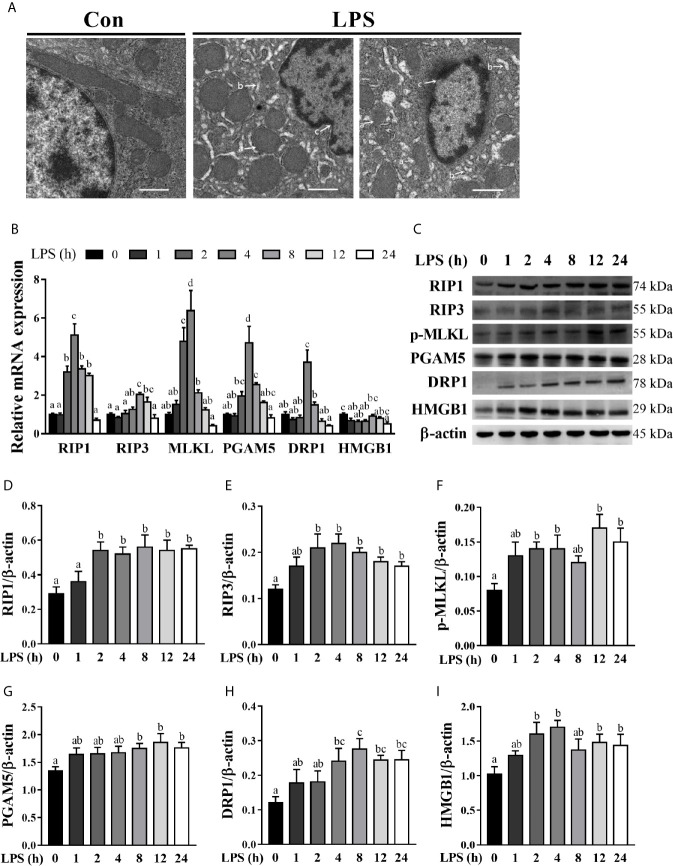
Hepatic cell necroptosis is dynamically induced in LPS-challenged piglets.The piglets in the control (Con) group were sacrificed at 0 h after injection with sterile saline, while those in LPS groups were sacrificed at 1, 2, 4, 8, 12, or 24 h after challenge with LPS at 100 μg/kg body weight. **(A)** Representative hepatic ultrastructures in Con and LPS groups. The liver sections were imaged using transmission electron microscopy. It is noted that mitochondrial swelling (a) and endoplasmic reticulum expansion (b) as well as nuclear deformation, nuclear membrane rupture, and chromatin overflow (c) were observed in LPS groups. Magnification: 5000 ×, scale bars = 1 μm. **(B)** mRNA abundance of necroptosis signaling components in the liver. **(C–I)** Protein abundance of necroptosis signaling components in the liver. Values are means ± SEM, n = 6. ^abcd^ Means without a common letter differ significantly (*p* < 0.05).

To explore if necroptosis occurred concomitant with hepatic injury and inflammation in LPS-challenged pigs, we examined liver ultrastructure by TEM (**Figure 3A**). Compared to the control pigs, LPS-challenged pigs exhibited significant hepatic ultrastructure damages showing mitochondrial swelling, endoplasmic reticulum expansion, nuclear deformation, nuclear membrane rupture, and chromatin overflow. To further explore the occurrence of necroptosis in septic liver, we measured the mRNA and protein levels of important components of necroptosis including RIP1, RIP3 and MLKL, PGAM5, DRP1, and HMGB1 (**Figures 3B–I**). As compared to the control group, LPS triggered a time-dependent up-regulation of RIP1, RIP3, MLKL, PGAM5, and DRP1 in the liver. Specifically, LPS increased the mRNA level of RIP1 between 2-12 h (peaking at 4 h and 5.12 fold), RIP3 between 8-12 h (peaking at 8 h and 2.04 fold), MLKL between 2-4 h (peaking at 4 h and 6.39 fold), PGAM5 between 4-8 h (peaking at 4 h and 4.72 fold) and DRP1 at 4 h (3.71 fold) (*p* < 0.05). However, LPS decreased mRNA abundance of HMGB1 at 1, 2, 4 and 24 h. Similarly, LPS elevated protein abundance of RIP1, RIP3 and phosphorylated MLKL between 2-24 h, and PGAM5 between 8-24 h and DRP1 between 4-24 h, peaking at 8, 4, 12, 12, 8 or 4 h, respectively (*p* < 0.05). Inconsistent with mRNA expression, protein abundance of HMGB1 was increased at 2, 4, 12 and 24 h (*p* < 0.05). These results, in combination with the severity of hepatic injury and inflammation, indicated that 4 h post LPS challenge is the time when the liver is experiencing dramatic changes and therefore, was chosen for analysis of the hepatic function and damage in a subsequent animal trial.

### Nec-1 Inhibits Hepatic Necroptosis in LPS-Challenged Piglets

**Figure 4:**
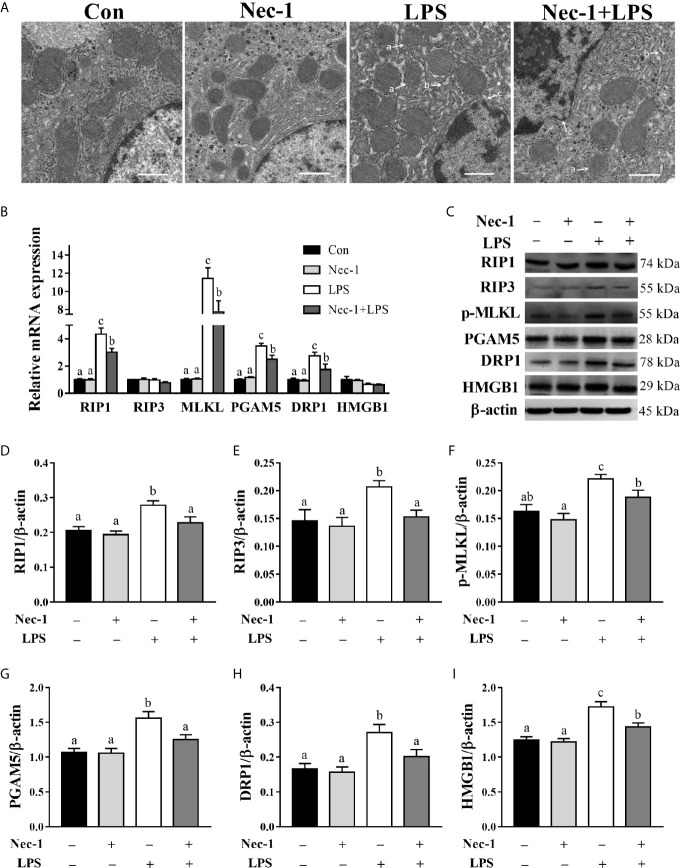
Necrostatin-1 (Nec-1) inhibits hepatic necroptosis in LPS-challenged piglets. Piglets were pretreated with Nec-1 at 1.0 mg/kg body weight or an equal volume of 2% DMSO 30 min before LPS or saline administration. Pigs were sacrificed at 4 h after LPS or saline injection. **(A)** Representative hepatic ultrastructural images by transmission electron microscopy. LPS caused significant necrosis such as mitochondrial swelling (a), endoplasmic reticulum expansion (b), nuclear deformation, nuclear membrane rupture and chromatin overflow (c), while Nec-1 markedly reduced the necrotic ultrastructural changes. Magnification: 5000 ×, scale bars = 1 μm. **(B)** mRNA abundance of necroptosis signaling components in the liver. **(C–I)** Protein abundance of necroptosis signaling components in the liver. Values are means ± SEM, n = 7. ^abc^ Means without a common letter differ significantly (*p* < 0.05).

To further demonstrate that LPS-induced hepatic damage is partially due to necroptosis, the piglets were pretreated with Nec-1, which is a specific inhibitor of RIP1 kinase activity (23) and RIP1/RIP3 association (11), 30 min prior to LPS challenge. TEM of the liver revealed that Nec-1 reduced LPS-triggered necrotic ultrastructural alterations (**Figure 4A**). LPS challenge for 4 h increased mRNA expression levels of RIP1, MLKL, PGAM5, and DRP1 and protein expression of RIP1, RIP3, phosphorylated MLKL, PGAM5, DRP1, and HMGB1 (*p* < 0.05) (**Figures 4B–I**). However, Nec-1 pretreatment reversed LPS-induced mRNA expression of RIP1, MLKL, PGAM5, and DRP1 as well as protein expression of RIP1, RIP3, phosphorylated MLKL, PGAM5, DRP1, and HMGB1 (*p* < 0.05).

### Nec-1 Alleviates Hepatic Damage, Dysfunction, and Inflammation in LPS-Challenged Piglets

**Figure 5:**
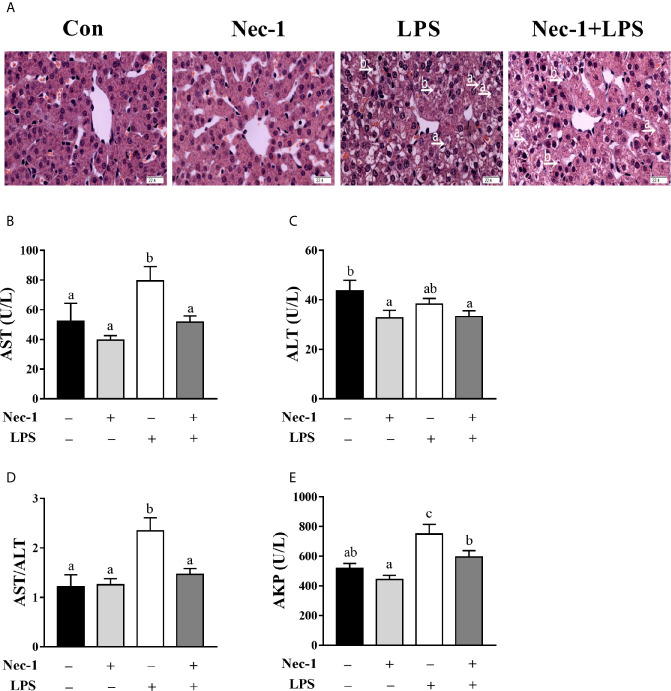
Necrostatin-1 (Nec-1) prevents hepatic morphological damage and dysfunction in LPS-challenged piglets. Piglets were pretreated with Nec-1 at 1.0 mg/kg body weight or an equal volume of 2% DMSO 30 min before LPS or saline administration. Pigs were sacrificed at 4 h after LPS or saline injection. **(A)** Representative haematoxylin/eosin-stained liver sections. LPS induced hepatic histopathological changes such as hepatocyte caryolysis (a) and karyopycnosis (b), disordered hepatic cell cord arrangement, and hepatocyte vacuolation, but was obviously alleviated by Nec-1. Magnification: 400 ×, scale bars = 22.4 μm. **(B–E)** Serum AST, ALT and AKP activities and the AST/ALT ratio. Values are means ± SEM, n = 7. ^abc^ Means without a common letter differ significantly (*p* < 0.05).

**Figure 6:**
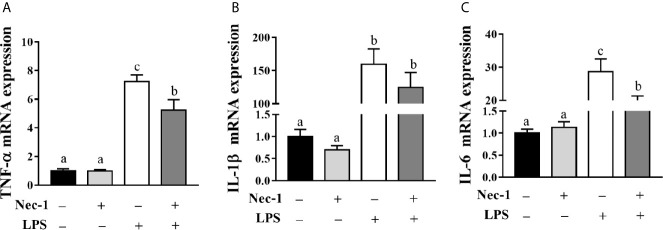
Necrostatin-1 (Nec-1) reduces hepatic inflammation in LPS-challenged piglets. Piglets were pretreated with Nec-1 at 1.0 mg/kg body weight or an equal volume of 2% DMSO 30 min before LPS or saline administration. Pigs were sacrificed at 4 h after LPS or saline injection. **(A–C)** TNF-α, IL-1β and IL-6 mRNA expression in the liver. Values are means ± SEM, n = 7. ^abc^ Means without a common letter differ significantly (*p* < 0.05).

We examined whether Nec-1 pretreatment could alleviate LPS-induced hepatic damage and dysfunction. Indeed, inhibition of necroptosis by Nec-1 obviously attenuated morphological damage in the liver 4 h post LPS challenge (**Figure 5A**). Desirably, Nec-1 largely restored the activities of AST, ALT, and AKP as well as the AST/ALT ratio in the serum of LPS-challenged piglets to basal levels (**Figures 5B–E**). Consistently, inhibition of necroptosis by Nec-1 significantly attenuated LPS-mediated induction of the mRNA expression levels of TNF-α and IL-6, but not IL-1β, in the liver (*p* < 0.05) (**Figures 6A–C**).

## Discussion

In addition to necrosis and apoptosis, a novel mode of cell death namely necroptosis was recently identified (8, 9). Necroptosis has similar morphological characteristics with necrosis, but is strictly controlled by an intracellular protein platform, similar to apoptosis (9, 10). Emerging evidence has indicated that necroptosis is implicated in the pathogenesis of alcoholic liver disease (16), non-alcoholic fatty liver disease (17), and ischemia-reperfusion injury of steatotic livers (18, 26). However, the involvement of necroptosis in septic liver injury is largely unknown.

In the current study, we evaluated the dynamic effect of LPS on hepatic damage in a piglet model. Hepatic injury has been found to be gradually increased up to 12 h post LPS challenge, and obviously diminished at 24 h. It is not surprising to observe concomitant hepatic dysfunction associated with hepatic histopathological changes. Serum AST and AKP activities as well as the AST/ALT ratio were evidently increased between 4-24 h post LPS challenge. These results are in agreement with our previous reports on a piglet model of LPS-induced sepsis; however, those studies only studied a single time point (4 or 24 h) after LPS challenge (22, 24).

Hepatic injury is closely associated with hepatic inflammation (22, 24). As expected, LPS evoked a marked increase of several representative pro-inflammatory cytokines such as TNF-α, IL-6 and IL-1β at both mRNA and protein levels. TNF-α, IL-6, and IL-1β mRNA expression levels in the liver were quickly peaked at 1 h after LPS challenge, while their protein levels were peaked within 4 h. The temporal patterns of LPS-induced changes of these cytokines are consistent with a previous observation in rats (27).

Given the fact that necroptosis is closely related to tissue injury and inflammation (15), we explored whether necroptosis occurs during LPS-induced hepatic damage. TEM analysis of the ultrastructure of hepatocytes damaged by LPS has revealed mitochondrial swelling, endoplasmic reticulum expansion, nuclear deformation, nuclear membrane rupture, and chromatin overflow, all of which are consistent with the characteristics of necrosis. A time-dependent increase in mRNA or protein expression levels of several critical components of necroptosis including RIP1, RIP3, MLKL, PGAM5, DRP1, and HMGB1 suggests the occurrence of necroptosis in the liver of LPS-challenged piglets. In agreement with our results, Shao et al. (28) showed that RIP1 protein was increased in a time-dependent manner in the brain cortex of LPS-challenged rats. In addition, Wang et al. (16) reported that LPS increased protein expression of RIP3, phosphorylated RIP3, and MLKL in the mouse lung 24 h post injection. Our results suggest that necroptosis may be induced quickly after LPS challenge and contribute to hepatic injury during sepsis.

To confirm the involvement of necroptosis in LPS-induced hepatic injury, Nec-1, a specific inhibitor of the RIP1 kinase activity and RIP1/RIP3 association (11, 23, 29), was used to inhibit necroptosis. Alleviation of ultrastructural hepatic necrotic changes by Nec-1 suggests that necroptosis is involved in LPS-induced liver injury. In addition, Nec-1 is capable of reducing the expression of RIP1 and MLKL as well as PGAM5, DRP1, and HMGB1 at mRNA or protein levels in the liver of LPS-treated pigs. Consistent with the involvement of necroptosis in liver damage, Nec-1 has been found to attenuate intestinal ischemia/reperfusion-induced liver injury (30). Inhibition of RIP1 by RIPA-56 alleviates experimental non-alcoholic fatty liver disease in mice (17). In addition, Knocking out the *Rip3* gene prevents ethanol-induced liver injury in mice (31). Our results demonstrate that necroptosis is involved in hepatic damage during sepsis.

Furthermore, we found that Nec-1 pretreatment decreased the mRNA abundance of hepatic TNF-α and IL-6, suggesting that necroptosis contributes to hepatic inflammation. Similarly, Takemoto et al. (32) reported that Nec-1 ameliorated hepatic IL-1β, IL-6, IL-10, chemokine (C-X-C motif) ligand 1, and basic fibroblast growth factor expression in acetaminophen-induced acute liver failure in mice. Ni et al. (26) showed that targeting necroptosis by knocking out MLKL decreased hepatic neutrophil infiltration and inflammation in ischemia-reperfusion injury of murine steatotic livers. Our study demonstrates that necroptosis exacerbates inflammation in the liver of LPS-challenge piglets.

In our study, regretfully, the preformed experiments and obtained results were not in-depth enough, which might weaken their contributions in clinical medicine. Firstly, Nec-1 was used before the sepsis was induced (LPS challenge). It is unlikely that pretreatment with Nec-1 could be applied in clinical practice, which may limit the clinical use of Nec-1. Further research is needed to determine practical and effective clinical use regimens of Nec-1 after the sepsis. Secondly, only a single dose of Nec-1 was used in this study. In future studies, multiple doses of Nec-1 should be evaluated to determine the optimal dose and therapeutic window of Nec-1 for maximal hepatic protection during sepsis.

In summary, we demonstrate that LPS activates necroptosis in the liver, resulting in the damage of hepatic structure and function. Targeting necroptosis with Nec-1 alleviates LPS-induced hepatic injury. Therefore, necroptosis occurs and contributes to hepatic damage during sepsis.

## Data Availability Statement

The raw data supporting the conclusions of this article will be made available by the authors, without undue reservation.

## Ethics Statement

The animal study was reviewed and approved by the Animal Care and Use Committee of Wuhan Polytechnic University.

## Author Contributions

YL, C-AH, and GZ designed research. QX, JG, XL, YW, KX, XW, and HZ conducted research. YL, XL, and YW analyzed data. YL, DW, and QX wrote the paper. DW, C-AH, and GZ revised the manuscript. YL had primary responsibility for final content. All authors contributed to the article and approved the submitted version.

## Funding

This research was financially supported by the Project of National Natural Science Foundation of China (No. 31772615), the Project of Innovative Research Groups of the Natural Science Foundation of Hubei Province (No. 2019CFA015), and Wuhan Science and Technology Bureau (No. 2018020401011304).

## Conflict of Interest

The authors declare that the research was conducted in the absence of any commercial or financial relationships that could be construed as a potential conflict of interest.

## Abbreviations

AKP, alkaline phosphatase; ALT, alanine aminotransferase; AST, aspartate aminotransferase; BW, body weight; DAMP, damage-associated molecular pattern; IL, interleukin; DRP1, dynamin-related protein 1; LPS, lipopolysaccharide; MLKL, mixed-lineage kinase domain-like protein; Nec-1, necrostatin-1; PAMP, pathogen associated molecular pattern; PGAM5, phosphoglycerate mutase family member 5; RIP, receptor interacting protein kinase; TNF-α, tumor necrosis factor-α; HMGB1, high-mobility group box 1.
