# Supplementary material for: Necroptosis Underlies Hepatic Damage in a Piglet Model of Lipopolysaccharide-Induced Sepsis
Source: Front Immunol. 2021 Mar 12;12:633830. doi: 10.3389/fimmu.2021.633830 (PMC7994362; doi:10.3389/fimmu.2021.633830)
Supplement: Supplementary file 1 [file DataSheet_1.docx]

**Supplementary Materials**

**Table 1S Treatment groups of the first experiment**

| Groups | Treatments^1^ |
| --- | --- |
| 0 | Slaughtered right (0 h) after saline injection |
| 1 | Slaughtered at 1 h after LPS challenge |
| 2 | Slaughtered at 2 h after LPS challenge |
| 4 | Slaughtered at 4 h after LPS challenge |
| 8 | Slaughtered at 8 h after LPS challenge |
| 12 | Slaughtered at 12 h after LPS challenge |
| 24 | Slaughtered at 24 h after LPS challenge |

^1^ LPS, 100 μg/kg body weight

**Table 2S Treatment groups of the second experiment**

| Groups | Treatments^1^ | |
| --- | --- | --- |
| Control | Saline | DMSO |
| Nec-1 | Saline | Nec-1 |
| LPS | LPS | DMSO |
| LPS+Nec-1 | LPS | Nec-1 |

^1^ LPS, 100 μg/kg body weight; Nec-1, 1.0 mg/kg body weight. Piglets were killed at 4 h after LPS or saline injection.
